# Social media’s contribution to political misperceptions in U.S. Presidential elections

**DOI:** 10.1371/journal.pone.0213500

**Published:** 2019-03-27

**Authors:** R. Kelly Garrett

**Affiliations:** School of Communication, Ohio State University, Columbus, Ohio, United States of America; National Taiwan University, TAIWAN

## Abstract

There is considerable concern about the role that social media, such as Facebook and Twitter, play in promoting misperceptions during political campaigns. These technologies are widely used, and inaccurate information flowing across them has a high profile. This research uses three-wave panel surveys conducted with representative samples of Americans during both the 2012 and 2016 U.S. Presidential elections to assess whether use of social media for political information promoted endorsement of falsehoods about major party candidates or important campaign issues. Fixed effects regression helps ensure that observed effects are not due to individual differences. Results indicate that social media use had a small but significant influence on misperceptions about President Obama in the 2012 election, and that this effect was most pronounced among strong partisans. Social media had no effect on belief accuracy about the Republican candidate in that election. The 2016 survey focused on campaign issues. There is no evidence that social media use influenced belief accuracy about these topics in aggregate, but Facebook users were unique. Social media use by this group reduced issue misperceptions relative to those who only used other social media. These results demonstrate that social media can alter citizens’ willingness to endorse falsehoods during an election, but that the effects are often small.

## Introduction

On November 19, 2016, the *New York Times’* editorial board published a scathing critique of Facebook’s failure to stop the spread of falsehoods in the lead up to the 2016 U.S. Presidential Election [1]. The opinion piece is emblematic of concerns about the threat that social media pose to democracy by corrupting citizens’ perceptions of political reality. Implicit in this claim is an assertion that the effects of social media on citizens’ belief accuracy are large, in contrast to the more limited effects associated with older media systems [2].

**Fig 1:**
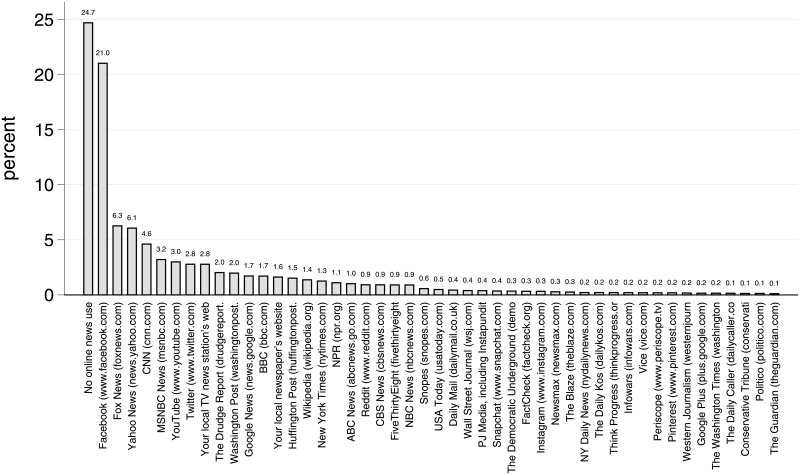
Site most frequently used for political information in past month. Data collected from a representative sample of Americans, *n* = 618, by the GfK Group in the weeks immediately following the 2016 election, using weights.

Reliance on social media for political news has increased rapidly. In 2012, about two in five Americans reported using social media for political purposes, and about one in three said they had encountered messages on social media promoting one of the candidates in the month leading up to the election [3, 4]. Four years later more Americans named Facebook as the site they most often used for political information in the month leading up to Election Day 2016 than named any other site, including those of high-profile news organizations such as Fox News, CNN, and major national newspapers (see Fig 1). This is troubling as online social networks have frequently been used to share political falsehoods, both about candidates and about important campaign issues [5–7]. Even more concerning, there is growing evidence that many of the falsehoods circulating during the 2016 U.S. Presidential Election were part of a Russian propaganda effort [8]. The idea that a foreign power would use social media in an effort to sow disinformation intended to sway an election is deeply troubling.

Exposure to deceptive messages is not tantamount to belief in them. Individuals often exhibit credulity, and act in ways intended to prevent themselves from being misled [9, 10]. Nonetheless, in a complex information environment, individuals’ cognitive limits and biases do make them susceptible to (political) misinformation. People find messages to be more believable the more familiar those messages are [11], suggesting that repeated contact with falsehoods shared online will encourage their acceptance. More importantly, people are prone to believe messages that affirm their political viewpoint or identity [12–14] regardless of the strength of the evidence, which suggests that partisan falsehoods are particularly likely to take root.

Inaccurate beliefs threaten the foundations of democracy [15]. Citizens shape the social and political environment through their engagement with politics, and especially through their participation in elections. Falsehoods undermine democratic processes by distorting decision making. Support for a candidate or policy depends fundamentally on what one believes, and falsehoods can color individuals’ judgments, potentially leading them to support positions that run counter to their self-interests [16]. Of course, promoting inaccurate claims about candidates and issues is only one of many communication strategies used to manipulate voters. Advancing conspiracy theories, which weave together truth and fiction in ways that appear to justify attributing social phenomena to a small but powerful group of self-interested individuals, are a related approach [17]. Accurate information can also be used strategically, as when confidential communication is leaked (often selectively) in order to cast a political opponent in a negative light [18]. This article, however, is focused on endorsements of candidate and issue falsehoods.

Attributes of the technological environment could exacerbate the effects of deceptive political messaging. Empirical evidence suggests that email, which like social media tends to follow the contours of existing interpersonal relationships, promoted rumor acceptance in a prior election [19]. Exposure to partisan online news, which is frequently shared via social media, has also been shown to contribute to misperceptions [20]. The very “socialness” of social media can make individuals less likely to verify the information they encounter online [21]. Patterns of content engagement are also potentially damaging. Social media users can be divided into highly segregated communities based on what they choose to like, comment on, or share [22]. This does not mean that users in these communities never see information challenging their viewpoint [23], but the behavior pattern is harmful nonetheless. For example, the resulting segregation could undermine efforts to correct falsehoods circulating on social media because individuals are more responsive to corrections from those with whom they regularly interact than from strangers [24].

There are, however, reasons to expect social media’s effects to be more limited. Survey research conducted immediately after the 2016 U.S. Presidential election estimated that the average American likely saw only a handful of verifiably false news stories during the campaign season, and that many who saw these messages were at least somewhat selective about which they believed [25]. Observational data collected during the campaign season suggest that ‘fake news’ was shared relatively rarely, and was concentrated among small subpopulations [26]. Although there were times when falsehoods were shared more often than real news [6], it is important to recognize that political information typically has a much smaller reach than other types of content [27], which could lessen the risk of harm.

Limits on media systems’ ability to alter citizens’ attitudes and beliefs are well known. Concern about citizens’ susceptibility to war-time propaganda and rumoring motivated an extensive body of social science research beginning in the 1940s [28] and continuing to the present day. This literature suggests that citizens use a host of strategies to protect and reinforce their predispositions, including being selective about the information to which they expose themselves [29], and engaging in ideologically motivated interpretations of evidence encountered [14]. Indeed, how individuals respond to messages to which they are exposed is often contingent on their political predispositions. Politically palatable claims are more readily accepted than claims that are less compatible with their political worldview. Thus, media effects often vary by users’ political affiliation. Media are still influential, but their effects tend to be small and contingent on host of other factors. The conclusion that media effects are limited still holds today [2], which is not to say that they are unimportant. Even small changes in belief accuracy can have consequential downstream effects on political behavior, including vote choice [30].

This project brings empirical evidence to bear on the question of what influence social media use has on Americans’ political belief accuracy and whether these effects are contingent on users’ partisan identity or which social media platforms they use. Panel data collected during the 2012 and 2016 U.S. Presidential elections from representative samples of Americans suggest that the limited media effects paradigm persists in the face of these new technologies. Fixed effects regression conducted with longitudinal data are used to estimate the effect of social media use on political belief accuracy, controlling for all stable individual differences. These regression models are extended through the addition of interactions terms to test several likely moderators.

## Data and analytic approach

Data come from a pair of three-wave panel surveys conducted during the 2012 and 2016 U.S. Presidential Elections. In each election, a large, representative, general population sample of Americans responded to the same set of survey questions at three points during the election cycle. Surveys in both elections repeat measures of social media use and belief accuracy in each wave, but they differ in terms of the misperceptions assessed. The first focused on candidate misperceptions, and the second on campaign issue misperceptions. The goal was to better understand whether and how social media effects would differ across these two important types of falsehoods.

The GfK Group collected the data, drawing respondents from its KnowledgePanel, a general population panel recruited using address-based sampling methods. The Ohio State University Behavioral and Social Sciences Institutional Review Board approved the study (Study Number 2012B0247). Consent was given digitally, via online consent forms.

The 2012 baseline data were collected July 13-August 6, immediately after the major party conventions (*N* = 1,004). Wave 2 was conducted August 31-October 3 (*N* = 783; 79.2% completion rate). The final wave was fielded November 2-19, immediately after the election (*N* = 652; 84.3% completion rate). The 2016 baseline data were collected between July 29 and August 11, and included 965 valid respondents (62% completion rate). 763 (81% completion rate) completed wave 2, which was fielded September 14-22. The third wave included 625 valid respondents (83% completion rate) and was collected November 9-14. Participant demographics for both surveys were comparable to the U.S. population (see Tables A and B in S1 File for more information). Neither social media use nor belief accuracy significantly influenced attrition.

Data are analyzed using fixed effects regression, a statistical technique that greatly improves researchers’ ability to make causal inferences using panel data despite a lack of random assignment [31, 32]. Fixed effects models are widely recognized as an invaluable tool for assessing panel data in the social sciences [33]. By analyzing within-respondent changes over time, these models treat unobserved variables as stable parameters, controlling for the influence of unmeasured factors in the resulting estimates. Model coefficients can be understood as describing the change in an outcome variable corresponding to a one-unit change in the predictor holding constant all stable characteristics of each respondent. Although it is not possible to estimate the effects of time-invariant variables when using this statistical technique, we can test whether the effects of time-varying variables are conditioned on stable parameters through the use of interaction terms.

Significance testing is conducted using 90% confidence intervals for predictions about candidate beliefs given prior evidence suggesting that social media promote candidate misperceptions. Tests use 95% confidence intervals for campaign beliefs based on the greater uncertainty about the direction of the relationship. All confidence intervals are estimated using 10,000 bootstrapped samples.

## Study 1: 2012 election candidate misperceptions

The 2012 survey focused on Americans’ endorsement of false political statements about candidates following other studies in the literature on misperceptions [5, 19]. Misleading claims about candidates are widespread, and the personal nature of the attacks may make them uniquely effective campaign tools. The falsehood endorsement measure assessed respondents’ beliefs in eight falsehoods, four critical of each of the two major-party candidates, Barack Obama (D) and Mitt Romney (R) (see Table 1). Several dozen candidate statements were collected with the assistance of Amazon Mechanical Turk workers, and the survey design team selected a subset that were unambiguously false based on the assessments of credible fact checkers such as Politifact, FactCheck, and major national news organizations. The final set of statements represents a range of issues and includes items that vary in terms of the media coverage they received. The belief battery also includes one true statement about each candidate.

**Table 1:** 2012 candidate belief accuracy.

Falsehood	Percent familiar with with rumor	Percent endorsing rumor^a^
Barack Obama is Muslim, not Christian.	90.8	29.0
Barack Obama is a Socialist because he believes the government should own the property and equipment used to produce goods.	58.0	30.8
Barack Obama said he wants gas prices to skyrocket so that Americans will switch to alternative energy sources.	51.8	40.4
Barack Obama used federal stimulus money to outsource U.S. bridge projects to Chinese companies.	50.3	41.3
As Governor of Massachusetts, Mitt Romneysigned a healthcare law providing taxpayer-funded abortions.	51.4%	51.5%
Mitt Romney, who is Mormon, does not call himself a Christian.	45.4	27.4
Mitt Romney said he knows what it means to work with the black community because his ancestors owned slaves.	32.2	34.0
Mitt Romney said that Mormon Church leaders should play a defining role in national affairs.	30.2	26.5

Based on wave 3 of 2012 survey (post-election), *n* = 652.

^a.^ Among those familiar with the rumor, who described it as “probably true” or “definitely true”.

Using an index of items with a range of prevalence helps to ensure that we are able to detect various types of social media influence over the course of the election. For example, use of these technologies might promote belief in widely discussed falsehoods, such as the claim that President Obama is Muslim, it might promote belief in more obscure inaccurate claims, such as the assertion that the President had outsourced federally funded construction work to foreign companies, or it might do both. This measurement approach should be able to detect a range of potential influences. Although these falsehoods were not equally widespread, data collected by Crimson Hexagon, a social media monitoring service, affirm that all eight claims were shared regularly on social media platforms such as Twitter and Reddit during the data collection period (Table C in S1 File). If citizens are being persuaded to adopt less accurate beliefs by their use of social media, these measures should detect the change.

In each wave, beliefs about the falsehoods were measured on a five-point scale, and recoded so that higher values denote greater accuracy (1 = Strong endorsement of falsehood, 3 = Ambivalence or uncertainty, 5 = Strong rejection). This measurement strategy means that it is possible to detect changes in belief confidence, even if a respondent’s beliefs do not convert from accurate to inaccurate (or vice versa). The four items for each candidate were averaged to create a composite belief accuracy score, and respondents who had never heard of any of the falsehoods about the target candidate prior to the survey were treated as missing in that wave (Obama: *M*s = 3.336 − 3.453, *SD*s = 1.204 − 1.248, *N*s = 604 − 893; Romney: *M*s = 2.762 − 3.212, *SD*s = 1.013 − 1.066, *N*s = 477 − 526). It is worth noting that there was considerable variance in respondents’ beliefs.

### Predictors

*Social media use* was measured in each wave of the panel using a series of self-reported behaviors. Individuals who said they used “an online social networking site, such as Facebook, Twitter, or LinkedIn” were asked how often they engaged in several behaviors: (a) following social media links to news sites, and consuming various types of content commonly found on social media, including (b) campaign news headlines, (c) campaign or candidate communications, (d) political opinions related to the election, and (e) election-related videos or images. Responses were given on a five-point scale, from “never” to “every day or almost every day,” and were recoded so that higher values correspond to more frequent use. (See Appendix A in S1 File for complete wording.) The five items were combined into a single composite measure (*M*s = 1.422 − 1.574, *SD*s = .761 −.958, *N*s = 651 − 1004; *alpha* = .925). It should also be noted that although the within-respondent changes in social media use are often modest, there are individuals who exhibit large wave-over-wave change (see Fig A in S1 File).

In order to assess whether social media’s influence varied by users’ *political affiliation*, respondents also reported their party affiliation in wave one on a seven-point scale, anchored by Strong Democrat (-3) and Strong Republican (3), with those choosing “something else” coded 0 (*M* = −.203, *SD* = 1.953). Statistical models include controls for other time-varying factors, including online and offline news use, and political email use (see Appendix A in S1 File for question wording and descriptives).

### Results

With cross-sectional data, scatterplots are typically used to assess between-respondent differences, comparing scores on two variables across the sample (see Figs B(1) and C(1) in S1 File). In contrast, a key advantage of panel data is that it allows us to assess within-respondent differences. In this case, a visual inspection of scatterplots comparing respondents’ change in social media use to their corresponding change in belief accuracy for each of the two candidates suggest that if there are significant relationships between these factors, their magnitude is small (see Figs B(2) and C(2) in S1 File). The fit line between changes in social media use and Obama belief accuracy between waves one and two has a negative slope, but between waves two and three it is flat. For Romney beliefs, the slope is nearly flat in both periods.

Linear fixed effects regression using the panel data provide a rigorous test of these relationships. These models estimate the effect of social media use on belief accuracy, controlling for use of other sources of political news and the passage of time as well as for any time-invariant individual differences. More specifically, estimates are calculated based exclusively on within-respondent differences over time, and the resulting estimates are unbiased by individual characteristics, despite the absence of control variables.

Estimates generated by random effects models risk being biased by individual differences; fixed effects models correct for this. A Hausman [34] test assesses whether the coefficients of a fixed effects model and of an equivalent random effects model that includes statistical controls are equal. Rejection of the null hypothesis indicates that estimates produced by the random effects model *are* biased by unmeasured between-respondent differences, and that a fixed effects model should be used. In this case, a Hausman test of the Obama beliefs model confirms that using fixed effects regression corrects for biases in a random effects model that are associated with unmeasured variables, *χ*^2^(6) = 15.87, *p* = 0.015. It is worth noting, however, that the random effects model coefficients (not shown) follow expected patterns among stable individual characteristics: Republicans tend to be less accurate than Democrats, while political interest and education are correlated with greater accuracy.

Fixed effects regression coefficients indicate that increasing social media use reduced respondents’ belief accuracy about Obama falsehoods, though the effect is small, 90% CI [−.120, −.003] (see Table D, model 1 in S1 File for the complete model). In the most extreme case, someone using social media to get political information in several different ways every day could have an accuracy score almost half a point lower than someone who did not use it at all, all other factors being equal.

Turning to the model of Romney belief accuracy, a Hausman test suggests that the more efficient random effects model is acceptable for this outcome variable, as it does not significantly bias coefficients, *χ*^2^(6) = 4.61, *p* = 0.595. Given this, predictions are tested using both a random effects model, to maximize efficiency, and a fixed effects model, for consistency with the Obama falsehood endorsement analyses. As with Obama falsehoods, the random effects model coefficients (not shown) for stable individual characteristics are as expected: Democrats tend to be less accurate than Republicans, while education is correlated with greater accuracy. Fixed effects model results indicate that although Romney beliefs became more accurate over time, the effect of social media was not significantly different than zero, 90% CI [−.104, .125] (Table E, model 1 in S1 File).

**Fig 2:**
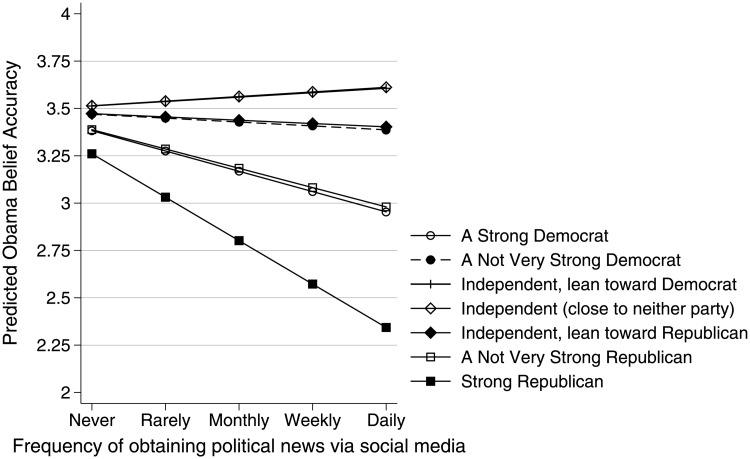
Candidate predictive margins. Estimated effect of social media use on candidate belief accuracy, by party affiliation.

The next pair of models look at whether social media’s effects vary by the user’s party affiliation. Individuals’ willingness to accept worldview-compatible falsehoods is well documented, and social media could exacerbate this tendency. Visual inspections of the data (not shown) suggest two patterns. As would be expected, it appears that Republicans tend to hold less accurate beliefs about President Obama than Democrats. There are also some indications that strong partisans on both sides tend to be less accurate than individuals taking a more moderate political stance. A model including interactions between social media use and both linear and quadratic terms representing party affiliation tests these relationships. The quadratic term is significant when modeling Obama belief accuracy, indicating that strength of partisanship amplifies the harmful effects of social media, 90% CI [−.035, −.007] (see Fig 2 and Table D, Model 2 in S1 File). The linear term is negative but non-significant: the difference in effect for Democrats and Republicans is indistinguishable from chance.

There is no evidence that the influence of social media use on Romney belief accuracy is contingent on users’ political affiliations (Table E, model 2 in S1 File).

## Study 2: 2016 election issue misperceptions

The 2016 analyses focus on four high-profile campaign issues, using a single measure of belief accuracy for each topic (see Table 2) at three points in time. Like candidate falsehoods, false claims about campaign issues can also promote inaccurate beliefs. Candidate falsehoods, however, tend to have a relatively low profile on the campaign trail. Candidates have historically avoided making unsubstantiated claims about their opponents a central part of their public campaigns—though whisper campaigns are not uncommon. Political issues, in contrast, tend to have a much higher profile. Candidates regularly use evidence in misleading ways in order to advance their political agenda [35]. Misleading claims about campaign issues can influence voters, altering their perception of, or support for, a policy decision. For example, beliefs about the economic impact of repealing the Affordable Care Act (ACA) would be expected to shape support for the policy. How citizens respond to such claims, however, could differ from their response to more personal attacks on candidates. Assessing social media’s influence on both types of falsehoods gives us a fuller understanding of the technologies’ political consequences.

**Table 2:** 2016 campaign issue belief accuracy.

Statement	Percent endorsing falsehood^a^
Repealing the Affordable Care Act would reduce the national debt (1)—Repealing the Affordable Care Act would increase the national debt (5)	32.6%
Most Muslims support violence against Western countries, including the U.S. (1)—Most Muslims oppose violence against Western countries, including the U.S. (5)	17.8
Immigrants are more likely to commit violent crimes than individuals born in the U.S. (1)—Immigrants are less likely to commit violent crimes than individuals born in the U.S. (5)	17.2
Human activity is contributing to changes in the global climate (5)—Human activity has no influence on global climate (1)	14.8

Based on wave 3 of 2016 survey (post-election), *n* = 623.

^a.^ Responses scored from 1 (most inaccurate) to 5 (most accurate), with scores of 1 or 2 coded as falsehood endorsement.

More than a dozen possible issues were identified, and the final set was selected based on frequent references to them on the campaign trail, extensive media coverage, and evidence that Americans were at least occasionally mistaken about them. This approach is considerably different than the one used when selecting candidate falsehoods. This was purposeful, and reflected differences in how the two types of falsehoods tend to be used during campaigns. Issue falsehoods are much easier to identify than false claims about candidates because they are more often openly embraced on the campaign trail, and are more widely reported in the news media. The question here is whether social media use is amplifying or constraining citizens’ willingness to endorse these widely shared falsehoods. These are not the only issues that were described inaccurately on the campaign trail, but they are among the highest profile claims. If social media were systematically promoting political falsehoods, we should see evidence of it here. Ground truth was determined based on evidence from a host of issue-relevant sources, including major news organizations (e.g., national newspapers and fact checking sites), scientific bodies (e.g., NASA), governmental agencies (e.g., the Congressional Budget Office), and non-partisan research organizations (e.g., Pew Research). Ambiguous issues were dropped.

In each wave, beliefs about each falsehood were measured on a five-point scale, and recoded so that higher values denote greater accuracy. The four issue beliefs were averaged to create a composite belief accuracy score (*M*s = 3.257 − 3.380, *SD*s = 0.812 −.844, *N*s = 624 − 936). As with candidate falsehoods, there is considerable variance in belief levels.

### Predictors

*Social media* use was measured in all three waves using a series of self-reported behaviors. Individuals who said they used at least one of 14 different social media services were asked how often they engaged in behaviors that included (a) reading political news headlines or summaries; (b) reading full news stories, not just summaries; (c) reading messages from political candidates, leaders, or organization; (d) reading other users’ comments on political news stories; (e) reading other users’ opinions about politics; or (f) looking at political videos or images. (See Appendix B in S1 File for complete wording.) The items use the same five-point response scale used in 2012, and they are combined into a single composite measure (*M*s = 1.940 − 2.102, *SD*s = 1.167 − 1.252, *N*s = 625 − 949; *alpha* = .966). As in 2012, change in social media use was often small, but there was heterogeneity across the sample (see Fig D in S1 File).

In contrast to 2012, the 2016 survey also asked respondents to identify which social media platform(s) they used in each wave. It included a battery of items modeled on the program-list technique, asking respondents to indicate with a check mark any sites or services they had used for news and information in the past month [36]. Facebook was one of over a dozen social media platforms listed, and these were embedded in a longer list of online news services (see Appendix B in S1 File). A dichotomous variable is used to indicate whether or not this service was selected. More than two in five Americans named Facebook as a news source in the prior month in each of the three survey waves.

*Party affiliation* was assessed in wave one with the same question that was used in 2012 (*M* = −.044, *SD* = 1.975). Statistical models also include controls for other time-varying factors, including online and offline news use, and political email use (see Appendix B in S1 File for question wording and descriptives).

As with the analysis of candidate misperceptions, the use of fixed effects regression with these panel data precludes the need for separate controls for stable individual differences.

### Results

A visual inspection of scatterplots suggests that the relationship between social media and belief accuracy is modest, both when viewed cross-sectionally and when comparing changes in the two factors (see Fig E in S1 File). There appears to be a slight upward trend in the relationship, raising the possibility that social media might improve belief accuracy. A random effects model (not shown) indicates that stable characteristics, which cannot be estimated with fixed effects models, behave as expected: Republicans tend to be less accurate than Democrats, consistent with the fact that the selected falsehoods were a prominent part of the Republican campaign strategy, while more educated individuals are more accurate.

A Hausman test indicates that the fixed effects approach is more appropriate, however, because random effects model coefficients are biased by unmeasured stable individual differences, *χ*^2^(8) = 59.69, *p* < 0.001. A fixed effects model estimating the influence of social media use on accuracy, controlling for other time-varying factors, provides no evidence that the technology measurably altered campaign issue belief accuracy (see Table F, Model 1 in S1 File).

**Fig 3:**
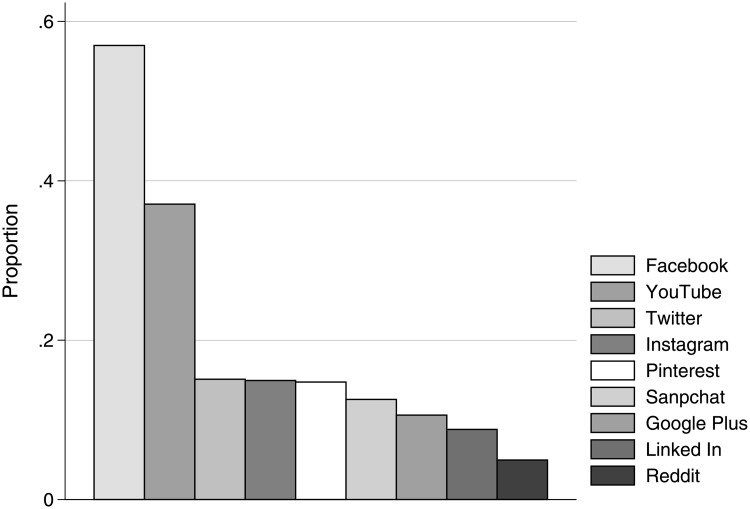
Platforms used to get news. Proportion of social media users who got news from popular platforms in the month leading up to the election.

The effect of social media, however, may be contingent on at least two other factors. First, it is possible that, as anticipated with candidate falsehoods, the effect of social media use depends on the user’s party affiliation. Second, given the different capabilities and practices associated with different social media platforms, the effect of the technology may be platform dependent. Facebook is by far the most widely used service, but many other social media platforms were used to get political information during the 2016 election season, including Twitter, YouTube, and Reddit (see Fig 3 and Table G in S1 File). It is worth noting that Facebook users got political news from social media more often than those who only used other social media platforms, *p* <.001 in all three waves). Individuals who did not use Facebook report almost no political news exposure via social media (*M* = 1.256, *SD* = 0.697), while those who did use it reported seeing political information regularly (*M* = 2.938, *SD* = 0.953). Furthermore, misinformation circulating on Facebook had a relatively high profile [6]. These factors make it important to understand whether Facebook had effects that are different from other social media platforms.

**Fig 4:**
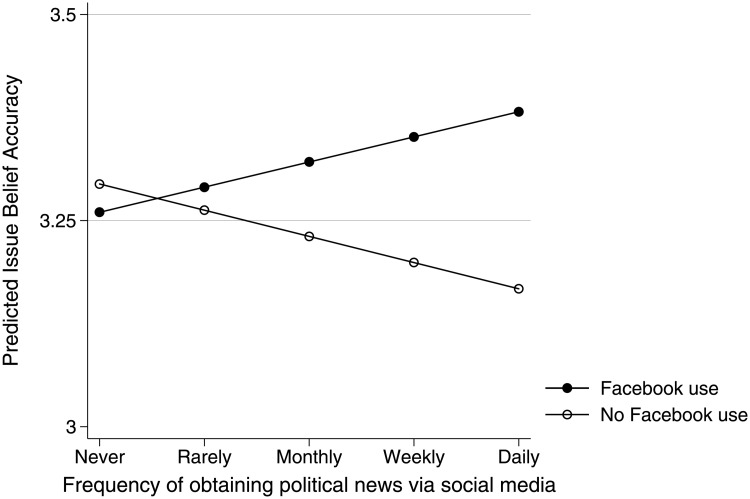
Campaign issue predictive margins. Estimated effect of social media use on issue belief accuracy, by Facebook use.

There is no evidence that party affiliation moderated social media’s influence (see Table F, Model 2 in S1 File). Facebook, however, does appear to be unique among social media platforms. Results suggest that the relationship between social media use and belief accuracy was more positive, 95% CI [.006 and.118], among Facebook users than among those who did not use the ubiquitous service. Among the heaviest users of social media for political news, individuals who used Facebook are expected to be almost a half point more accurate, on average, than those who only used other social media platforms, all else equal (see Fig 4). The magnitude of this effect is small, but it does call into question the presumption that Facebook had a uniquely harmful influence, at least when considering campaign issue beliefs. It should be noted, however, that this test is made weaker by the fact that only about one in five respondents started or stopped using Facebook in each wave (see Fig D in S1 File.

## Discussion

These data suggest that social media have a small but significant influence on Americans’ belief accuracy. Heavier use of these communication services was associated with a slight increase in the likelihood of endorsing falsehoods about President Obama in 2012, but it had no effect on beliefs about the Republican candidate. In 2016, social media use had no measurable aggregate influence on issue beliefs, but frequent use by Facebook users did more to promote accuracy than use by those who only interacted with other social media platforms during the election season. The evidence that social media can play a role in shaping Americans’ political beliefs is important. At the same time, though, the idea that social media are not a major driver of political misperceptions stands in contrast to numerous popular media accounts. Importantly, these empirical results are consistent with other studies of the role of social media on belief accuracy, which suggest that exposure to ‘fake news’ was limited [26, 37, 38] and that the influence on beliefs was quite modest [25]. There is no question that a large number of falsehoods circulated during the past two U.S. Presidential elections, and that millions of Americans have engaged with inaccurate messages via social media. This research suggests, however, that during the 2012 and 2016 U.S. presidential elections, social media use contributed relatively little to Americans’ willingness to endorse political falsehoods.

The tests used here are rigorous. They rely on panel data and fixed effects regression to help ensure that relationships are not due to unmeasured individual differences. However, given the size of the sample, and the one-tailed significance tests used when modeling candidate beliefs, it is unlikely that substantively important effects are being overlooked. It may be that social media use has a larger effect among some subpopulations, that its effects in one group counteract its effect in another, or that there are other falsehoods not assessed here for which the technology is more influential. These possibilities merit further consideration. Still, the average treatment effect is itself important. There is simply no evidence that social media are having a powerful and consistent influence on citizens’ belief accuracy.

That social media affect users, but that the aggregate effects are of limited magnitude, suggests that despite the many novel characteristics of the technologies, the way that people respond to messages delivered over social media is not so different from how they respond to other communication media. Indeed, social science research dating back to the 1940s has shown that media effects are often limited [39], and most research suggests that this pattern persists today [2]. There are a variety of ways that people maintain their attitudes and beliefs in an information environment that could challenge them. Individuals are selective about what they consume [29, 40] and cautious about what they believe [13] at least some of the time.

Studies of misperceptions often draw attention to the negative implications of resistance to factual corrections, as when citizens refuse to update their beliefs in the face of overwhelming evidence (e.g., climate change, Obama’s birthplace, WMDs in Iraq). The same mechanisms that drive this resistance, however, can also help individuals avoid being deceived by misinformation circulating on social media. The practice of scrutinizing especially carefully claims that run counter to existing beliefs does produce bias, undermining individuals’ ability to update their beliefs in light of new evidence. At the same time, though, it makes it less likely that people will revise their beliefs based on implausible claims. This may help to explain many social media users’ skepticism toward the falsehoods in circulation during the 2016 election [41].

The fact that these effects are small does not mean that they are unimportant. Accurate assessments of the political information environment are critical to citizens’ success in voting in their own interest [15, 16], and sowing doubt about empirical evidence can be a powerful political strategy [42]. In an electoral context, increased willingness to endorse political falsehoods can have important consequences for citizens’ subsequent vote choice [30]. When election margins are small, even small differences can be decisive. Furthermore, it is likely that there are subsets of the population for whom these effects are larger. For example, traffic to ‘fake news’ sites in 2016 was driven in large part by individuals whose overall news diets were highly conservative [38] making stronger effects among this group more likely.

The results summarized here do not, however, explain how political misperceptions have come to be a hallmark of the contemporary political environment. Political disagreement is eternal, and there has always been contention over what constitutes political truth [43], but in the U.S. political system there have been periods when citizens were in greater accord about claims of fact. Science offered important insights that could inform decision making; and while the significance of empirical evidence might be disputed, its existence was not. The willingness of millions of Americans to conclude that science is corrupt, that economic data and financial models are untrustworthy, and that journalists and fact checkers serve a single-minded political purpose is a threat to our democracy.

The value of this study lies in challenging assumptions, encouraging scholars both to recognize the small but significant influence of social media and also to look for alternative mechanisms to explain the prevalence of political misperceptions in the U.S. today. If social media are not a primary driver of belief in falsehoods, what is? The results of this study suggest that we should turn more of our attention to other explanations.

### Limitations

These data have important limitations. First, panel data were collected over a period of several months, which is considerably shorter than many disinformation campaigns. Falsehoods about the birthplace of President Obama can be traced back to 2008; they were repeatedly raised by President Trump in the years that followed; and they were still being endorsed by Republican politicians as recently as 2017 [44]. Strategic efforts to undermine climate change have gone on for even longer [42]. Second, and related to the first point, many high profile falsehoods circulating during the 2016 election emerged late in the campaign season [6], and were rapidly promoted using automated accounts [45]. In other words, some disinformation campaigns appear designed to gain attention quickly before flaring out. The absence of belief measures immediately prior to Election Day, when campaign interest and involvement are especially high—and when misinformation on social media was shared most extensively in 2016—could contribute to the failure to detect large effects in these tests.

The research design used here is insufficient for assessing dynamics on either of these alternative time scales. Although the effects of social media over several months are small, perhaps the technology is promoting longer-term changes in citizens’ beliefs. A steady diet of falsehoods could chip away at citizens’ resistance, promoting misinformation and undermining faith in reliable information outlets. Nor do these data preclude the possibility that social media are promoting short-lived deception. It is not implausible, for instance, that some individuals believed that the Pope had endorsed Trump, at least briefly, after seeing the claim on Facebook. Data about both shorter and longer-term effects are an important complement to this research.

The 2016 data are also limited by their disproportionate focus on falsehoods that Republicans would be more likely to believe. This reflected the nature of the false claims circulating at the time the belief statements were drafted, but future work would do well to consider misperceptions more commonly held by Democrats.

### Social significance

The fight against political misperceptions is fundamental to democracy, but in order to create an effective response, we must understand the mechanisms that promote the acceptance of falsehoods. The best evidence that we have to date suggests that social media are influential, but that they are not a primary cause of falsehood endorsement. This is not an excuse for complacency with regard to social media. It is clear that these communication technologies are being used to promote misperceptions for political ends, and though the effects are small, small effects matter. Furthermore, there can be no doubt that attempts to use social media to deceive will become more sophisticated. Those who seek to advance political propaganda to promote their own interests may find new ways to turn technology to their advantage. More importantly, misperceptions are not the only threat posed by manipulations of social media. Many other types of problematic information are not considered here. Social media’s modest role in promoting endorsement of candidate falsehoods may, for example, be dwarfed by its capacity to promote conspiracy theories. The technology provides a unique opportunity for relatively small groups of individual to find one another, forming communities of like-minded individuals who work together to affirm their beliefs [46].

These results do, however, suggest that it may be valuable to broaden the focus of technological and political interventions. Disinformation campaigns are part of a larger democratic malaise, alongside increasing political polarization of emotions towards those we disagree with [47], strategic use of anger and incivility to promote political objectives [48, 49], and deteriorating faith in institutions intended to promote knowledge and understanding [50]. It may prove more effective to look for ways to help society resist these threats than to target disproportionately social media’s role in promoting misinformation.

## Conclusion

Over the past decade, social media, and especially Facebook, have transformed how Americans get political news. Perhaps as a consequence of their high profile, these technologies have become an important conduit over which misinformation is spread. Panel data collected during the U.S. Presidential elections in 2012 and 2016 suggest that despite the prevalence of falsehoods on these networks, their influence on citizens’ beliefs is relatively small. Social media use produced only a small increase in endorsement of falsehoods about President Obama, and had no effect on beliefs about his competitor in the 2012 election. Contrary to the conventional wisdom, in 2016 social media use had more positive (or less negative) effects on campaign issue belief accuracy among Facebook users than among those who did not use the social networking platform. Taken together, these results suggest that a single-minded focus on fighting misinformation on social media is shortsighted. There may be other, more important sources of misinformation, and social media may have other more important harmful effects on democracy.

## Supporting information

S1 File Supporting information. This file contains supporting information.
(PDF)
