# Supplementary material for: Social media’s contribution to political misperceptions in U.S. Presidential elections
Source: PLoS One. 2019 Mar 27;14(3):e0213500. doi: 10.1371/journal.pone.0213500 (PMC6436681; doi:10.1371/journal.pone.0213500)
Supplement: S1 File — This file contains supporting information. (PDF) [file pone.0213500.s001.pdf]

Supporting Information for

Social media's contribution to political misperceptions in U.S. Presidential elections

R. Kelly Garrett*

Ohio State University

* Corresponding author. Email: garrett.258@osu.edu

The file includes:

- Appendices A to B
- Tables A to G
- Figs A to E

Contents

Appendix A. Question wording and descriptives for 2012 election study 3
Appendix B. Question wording and descriptives for 2016 election study 4
Table A. Sample demographics for 2012 election study, Waves 2 & 3 6
Table B. Sample demographics for 2016 election study, Waves 2 & 3 6
Table C. 2012 candidate falsehood prevalence on social media 7
Table D. Fixed-effects models of 2012 Obama belief accuracy.. 8
Table E. Fixed-effects models of 2012 Romney belief accuracy 8
Table F. Fixed-effects models of 2016 issue belief accuracy.. 9
Table G. Proportion of social media users getting news on each platform, by wave....... 10
Fig A. Wave-over-wave change in social media use, 2012.. 11
Fig B. Scatterplots comparing social media use and Obama belief accuracy. 12
Fig C. Scatterplots comparing social media use and Romney belief accuracy. 13
Fig D. Wave-over-wave change in social media use, 2016.. 14
Fig E. Scatterplots comparing social media use and campaign issue accuracy............... 15

Appendix A. Question wording and descriptives for 2012 election study

Social media use was assessed in a two ways. First, a question asked "How often did you use the following strategies to get to the news sites that you used in the past month?" Several behaviors were listed, including "Followed a link posted on a social network, such as *Facebook* or *Twitter.*" Second, respondents who answered in the affirmative when asked if they had ever used "an online social networking site, such as Facebook, Twitter, or LinkedIn" were asked "How often did you use social networking sites to do the following in the past month? (a) Read news headlines or short news summaries about the Presidential candidates or the campaign; (b) read messages from, or profiles of, the presidential candidates or the campaign; (c) read political opinions about the presidential candidates or the campaign; (d) look at a video or image about the Presidential candidates or the campaign." Descriptives are reported in the main text of the manuscript.

Offline news use was assessed by asking respondents how often they get news or information from five offline sources in the past month: print newspapers, television, print magazines, radio news, and political talk radio. Responses are given on a five-point scale, from "never" to "every day or almost every day", and are recoded so that higher values correspond to more frequent use. The items were combined to form a single composite measure ($M = 3.432$, $SD = .862$, $alpha = .716$)

There were several measures of *online news use*. Partisan site use was assessed by asking respondents how often they got information about the election in the past month from the website of a major national news organization frequently characterized as favoring liberal (conservative) positions and the websites of a politically liberal (conservative) online news organization or blog. Measures of non-partisan site use asked about major news organizations not frequently characterized as favoring a particular party of ideology, and nonpartisan online news organizations and blogs. Responses are given on a five-point scale, from "never" to "every day or almost every day", and are recoded so that higher values correspond to more frequent use. The items were combined to form a single composite measure ($M = 1.527$, $SD = .688$, $alpha =$ 838).

Email use was assessed by asking respondents how often they read political emails from three sources in the past month: friends and family; political candidates, parties, or other groups; and news organizations. Responses are given on a five-point scale, from "never" to "every day or almost every day", and are recoded so that higher values correspond to more frequent use. The items were combined to form a single composite measure ($M = 1.715$, $SD = .888$, $alpha = .804$).

Appendix B. Question wording and descriptives for 2016 election study

Social media use was assessed in two stages. First, respondents indicated which of 14 different social media sites they had used at least once in the past month (listed in the Online news use subsection, below). Second, respondents who reported using any of those sites were presented with the following message: "You indicated that you use some type of social media. Though not always intentional, people do sometimes *encounter* news and information about politics, including the election, using these services. How about you? How often did you use social media to do the following **in the past month**? (a) Read political news headlines or short news summaries; (b) Access and read political news stories (not just headlines or summaries); (c) Read messages from, or profiles of, political candidates, leaders, or organizations; (d) Read other users' comments about political news stories; (e) Read other users' opinion about politics; (f) Look at a political photo, meme, video, gif, etc." Descriptives are reported in the main text of the manuscript.

Offline news use was measured as it was in 2012. The items were combined to form a single composite measure (M = 2.625, SD = .877, *alpha* = .760).

Online news use was based on the program list technique (Dilliplane, Goldman, & Mutz, 2013). Respondents first indicated whether they had learned about politics in the prior month from online sources, including news sites, blogs, news app, or from social media sites or apps, such as Facebook, YouTube, or Twitter. Those who had were presented with a list of online sources, in random order, that included 65 frequently used news sites of varying degrees of partisanship. Use was measured as a count of sites (M = 4.271, SD = 5.133). A complete list of news sites follows: Google News (news.google.com), Yahoo News (news.yahoo.com), CNN (cnn.com), Buzzfeed (buzzfeed.com), Washington Post (washingtonpost.com), USA Today (usatoday.com), Vice (vice.com), Daily Mail (dailymail.co.uk), Business Insider (businessinsider.com), BBC (bbc.com), The Guardian (theguardian.com), NBC News (nbcnews.com), NPR (npr.org), ABC News (abcnews.go.com), CBS News (cbsnews.com), Politico (politico.com), NY Daily News (nydailynews.com), LA Times (latimes.com), Gawker (gawker.com), Time (time.com), Wikipedia (wikipedia.org), FactCheck (factcheck.org), Politifact (politifact.com), Snopes (snopes.com), FiveThirtyEight (fivethirtyeight.com), New York Times (nytimes.com), Huffington Post (huffingtonpost.com), Slate (slate.com), The Daily Beast (thedailybeast.com), The Daily Kos (dailykos.com), The Atlantic (theatlantic.com), Vox (vox.com), MSNBC News (msnbc.com), Salon (salon.com), The New Yorker (newyorker.com), Talking Points Memo (talkingpointsmemo.com), Mother Jones (motherjones.com), Think Progress (thinkprogress.org), Alternet (alternet.org), Newsweek (newsweek.com), The Nation (thenation.com), The New Republic (newrepublic.com), MoveOn (moveon.org), The Democratic Underground (democraticunderground.com), Crooks and Liars (crooksandliars.com), Fox News (foxnews.com), Conservative Tribune (conservativetribune.com), Wall Street Journal (wsj.com), The Drudge Report (drudgereport.com), Western Journalism (westernjournalism.com), New

York Post (nypost.com), Breitbart (breitbart.com), The Blaze (theblaze.com), Newsmax (newsmax.com), The Daily Caller (dailycaller.com), National Review (nationalreview.com), The Washington Times (washingtontimes.com), Young Conservatives (youngcons.com), PJ Media, including Instapundit (pjmedia.com), Infowars (infowars.com), The Weekly Standard (weeklystandard.com), Top Right News (toprightnews.com), Cybercast News Service (www.cnsnews.com), Right Wing News (rightwingnews.com), and Newsbusters (newsbusters.org).

Furthermore, for respondents who reported getting news from *social media*, fourteen social media platforms were included in the list of online news sources, in random order. These platforms were: Facebook (www.facebook.com), Google Plus (plus.google.com), LinkedIn (www.linkedin.com), Twitter (www.twitter.com), Tumblr (tumblr.com), Reddit (www.reddit.com), Pinterest (www.pinterest.com), YouTube (www.youtube.com), Vine (vine.co), Instagram (www.instagram.com), Snapchat (www.snapchat.com), Periscope (www.periscope.tv), Meerkat (meerkatapp.co) , Yik Yak (www.yikyak.com)

Email use was assessed by asking respondents how often they read political emails in the past month from either their friends and family, or anyone else. The items used the same five-point response scales as were used in 2012, from "never" to "every day or almost every day", and were coded so that higher values correspond to greater frequency. The items were combined to form a single composite measure (M = 1.544, SD = .731, r = .665).

References

Dilliplane, S., Goldman, S. K., & Mutz, D. C. (2013). Televised Exposure to Politics: New Measures for a Fragmented Media Environment. *American Journal of Political Science, 57*(1), 236-248. doi: 10.1111/j.1540-5907.2012.00600.x

Table A. Sample demographics for 2012 election study, Waves 2 & 3

	Wave 2	Wave 3
Gender (female)	50.00%	49.69%
Age	M = 50.64 (SD = 16.23)	M = 50.85 (SD = 16.07)
Education		
Less than high school	8.44%	7.98%
High school	29.80	30.06
Some college	26.21	26.07
Bachelor's degree of more	35.55	35.89
Party		
Democrat	45.34%	45.74%
Independent or other party	18.78	18.14
Republican	35.88	36.12
Income	Mode = $100,000, Median = $60,000	Mode = $100,000, Median = $50,000
Race/ethnicity:		
White, Non-Hispanic	76.09%	76.99%
Hispanic	9.72	8.74
Black, Non-Hispanic	7.93	7.67
Other	6.27	6.60

Note: Respondents had to participate in at least two consecutive waves to be included in the analysis.

Table B. Sample demographics for 2016 election study, Waves 2 & 3

	Wave 2	Wave 3
Gender (female)	50.98%	47.36%
Age	M = 49.59 (SD = 17.73)	M = 50.27 (SD = 17.59)
Education		
Less than high school	8.65%	8.32%
High school	26.47	25.12
Some college	29.36	29.60
Bachelor's degree of more	35.52	33.96
Party		
Democrat	39.31%	38.42%
Independent or other party	22.69	20.42
Republican	37.99	38.42
Income	Mode = $100,000, Median = $60,000	Mode = $100,000, Median = $60,000
Race/ethnicity:		
White, Non-Hispanic	74.31%	75.20%
Hispanic	10.48	10.56
Black, Non-Hispanic	8.39	7.20
Other	6.82	7.04

Note: Respondents had to participate in at least two consecutive waves to be included in the analysis.

Table C. 2012 candidate falsehood prevalence on social media

Keywords	Mentions
Obama Muslim	190,657
Obama Socialist	82,376
Obama Gas Skyrocket	729
Obama Outsource China Chinese	113
**Obama Total**	**273,875**
Romney Not Christian	3,770
Romney Ancestors Slaves	3,108
Romney Mormon Leaders	438
Romney Abortion Tax Funded	36
**Romney Total**	**7,352**

Notes. Social media mentions on Twitter, Instagram, Reddit, Google Plus, Tumblr, and YouTube between June 1, which was about six weeks prior to fielding the survey, and November 19, the last day the survey was in the field.

Table D. Fixed-effects models of 2012 Obama belief accuracy

	Model 1	Model 2
Social Media Use (SM)	**(-.120, -.003)**	(-.063, .112)
SM X Party affiliation [a]	—	(-.047, .006)
SM X Party affiliation^2	—	**(-.035, -.007)**
Wave 2	**(.085, .185)**	**(.087, .187)**
Wave 3	**(.055, .164)**	**(.055, .166)**
Email use	(-.064, .040)	(-.064, .038)
Offline news use	(-.053, .075)	(-.057, .071)
Online news sites used	(-.080, .054)	(-.085, .050)
Constant	**(3.214, 3.648)**	**(3.231, 3.662)**
# of Observations	2,212	2,188
# of Groups	948	936
Prop. of DV variance due to fixed effects	.818	.814
R^2: within	.020	.025
R^2: overall	.001	.034

Notes. Cell values indicate 90% Confidence Intervals (CI) for coefficients based on 10,000 bootstrapped samples. a. Anchored by Strong Democrat (-3) and Strong Republican (3).

Table E. Fixed-effects models of 2012 Romney belief accuracy

	Model 1	Model 2
Social Media Use (SM)	(-.104, .125)	(-.111, .256)
SM X Party affiliation [a]	—	(-.062, .044)
SM X Party affiliation^2	—	(-.046, .013)
Wave 2	**(.051, .244)**	**(.053, .249)**
Wave 3	**(.285, .505)**	**(.287, .512)**
Email use	(-.058, .173)	(-.060, .170)
Offline news use	(-.096, .160)	(-.102, .158)
Online news sites used	(-.187, .079)	(-.191, .077)
Constant	**(2.326, 3.018)**	**(2.349, 3.043)**
# of Observations	1,523	1,510
# of Groups	765	758
Prop. of DV variance due to fixed effects	.540	.539
R^2: within	.055	.056
R^2: overall	.029	.024

Notes. Cell values indicate 90% Confidence Intervals (CI) for coefficients based on 10,000 bootstrapped samples. a. Anchored by Strong Democrat (-3) and Strong Republican (3).

Table F. Fixed-effects models of 2016 issue belief accuracy

	Model 1	Model 2
Social Media Use (SM)	(-.024, .039)	(-.078, .014)
SM X Party affiliation [a]	—	(-.020, .010)
Facebook use?	—	(-.240, .047)
SM X Facebook use	—	**(.006, .118)**
Wave 2	(-.016, .068)	(-.014, .069)
Wave 3	**(.048, .144)**	**(.051, .146)**
Email use	**(.007, .093)**	**(.006, .095)**
Offline news use	(-.051, .039)	(-.050, .040)
Online news sites used	(-.010, .004)	(-.011, .003)
Constant	**(3.059, 3.347)**	**(3.089, 3.396)**
# of Observations	2,312	2,301
# of Groups	942	937
Prop. of DV variance due to fixed effects	.770	.759
R^2: within	.018	.022
R^2: overall	.000	.031

Notes. Cell values indicate 95% Confidence Intervals (CI) for coefficients based on 10,000 bootstrapped samples. a. Anchored by Strong Democrat (-3) and Strong Republican (3).

Table G. Proportion of social media users getting news on each platform, by wave

Platform	Wave 1	Wave 2	Wave 3
Facebook	56%	58%	57%
YouTube	36%	36%	37%
Twitter	14%	15%	15%
Instagram	15%	15%	15%
Pinterest	12%	15%	15%
Snapchat	13%	12%	13%
Google Plus	10%	10%	11%
Linked In	10%	11%	9%
Reddit	7%	5%	5%
N	731	590	501

Note. Data collected during 2016 election, using weights. Platforms used by less than 5% of social media users not shown.

Fig A. Wave-over-wave change in social media use, 2012

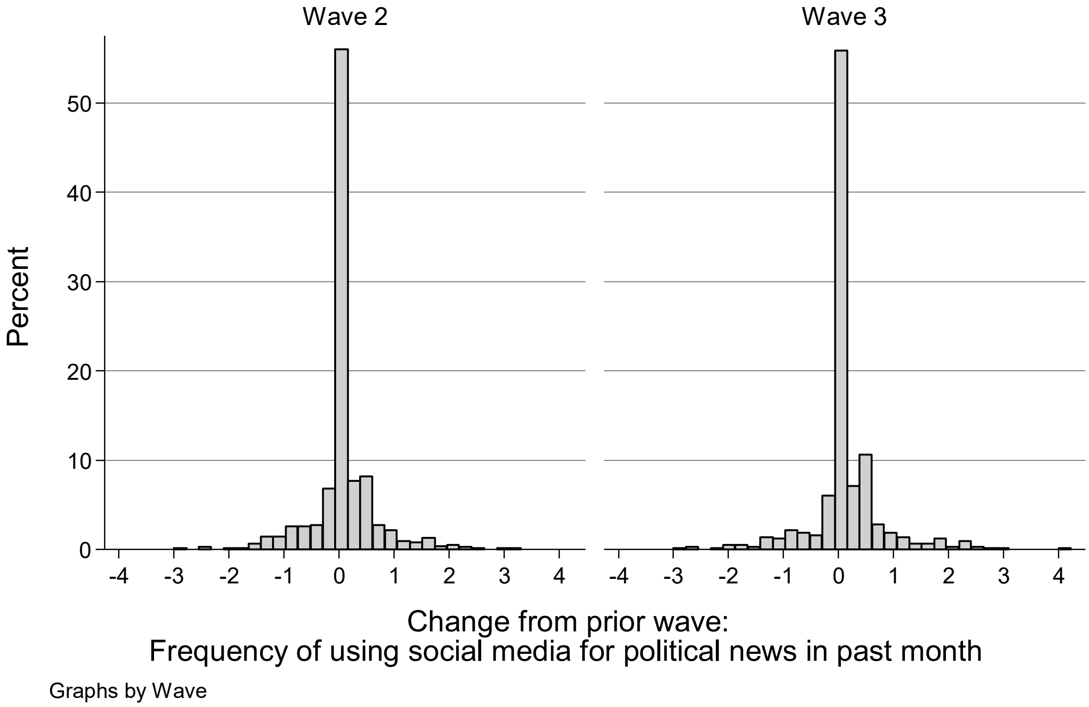

Note. Distributions of change in frequency of social media use from prior wave in 2012.

Fig B. Scatterplots comparing social media use and Obama belief accuracy.

(1)

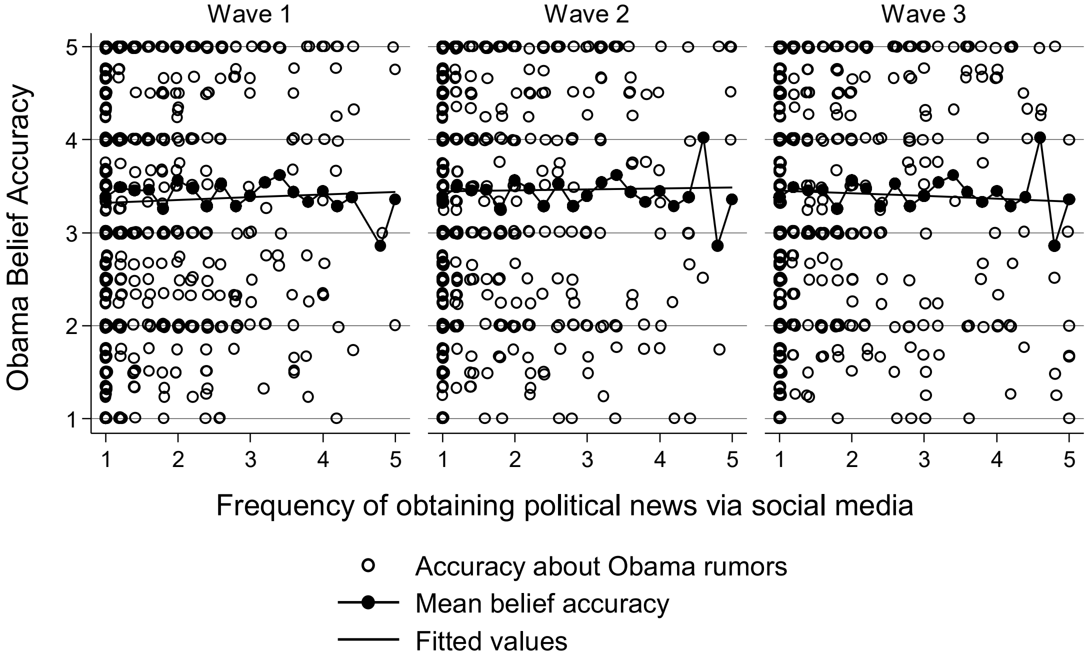

(2)

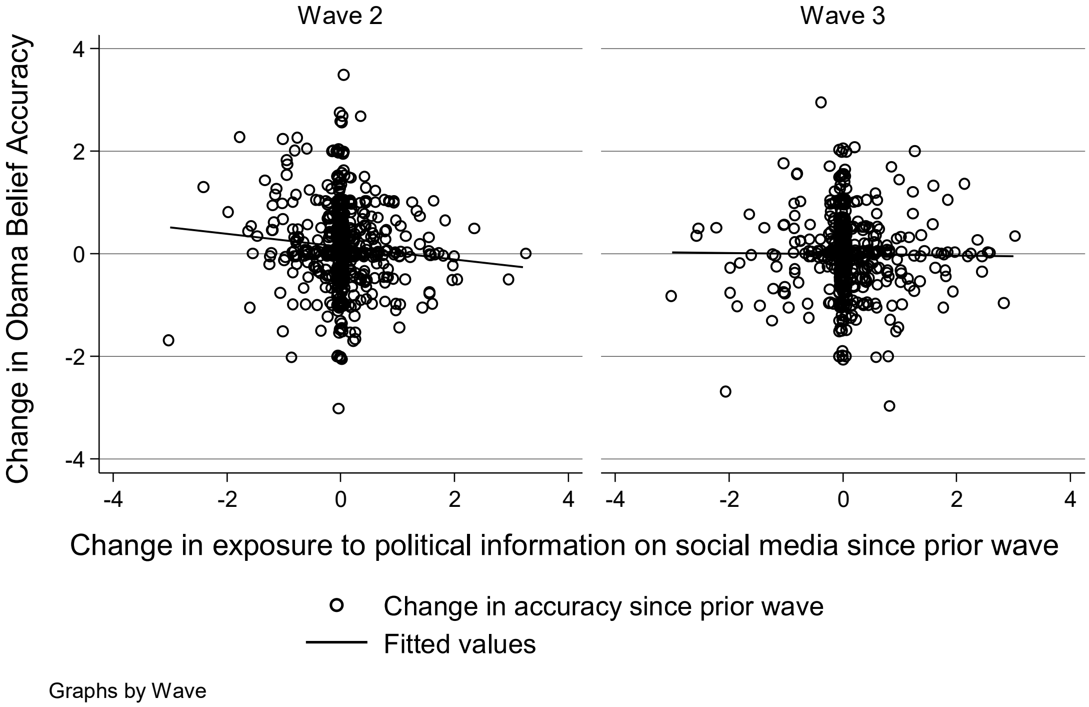

Note. Top figure (1) shows cross-sectional relationship, bottom figure (2) shows relationship between changes in social media use and belief accuracy.

Fig C. Scatterplots comparing social media use and Romney belief accuracy.

(1)

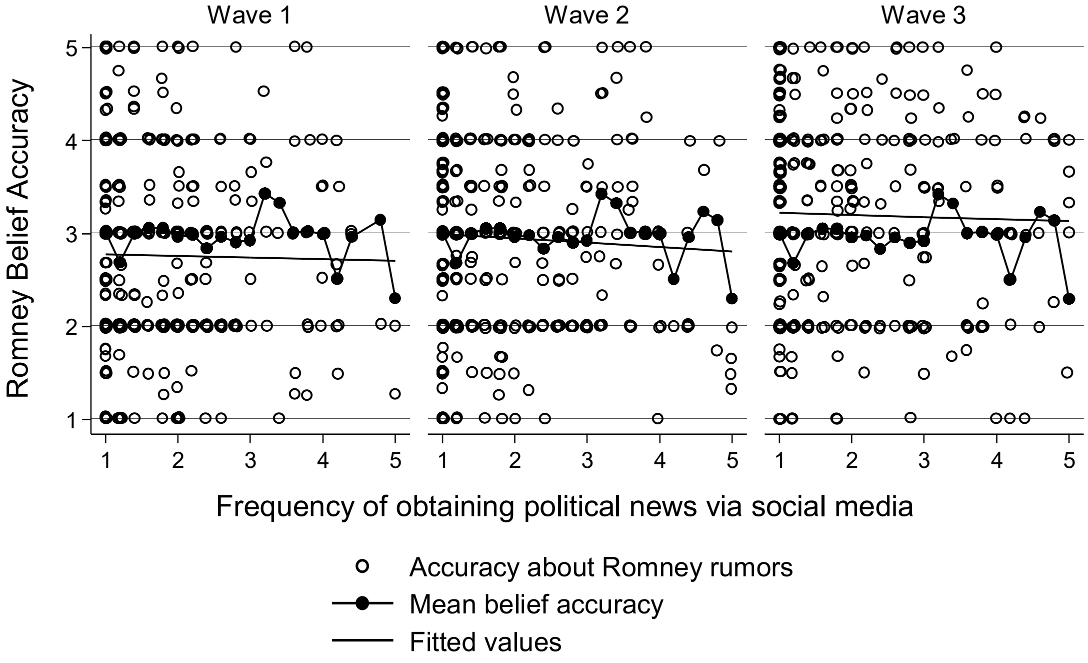

(2)

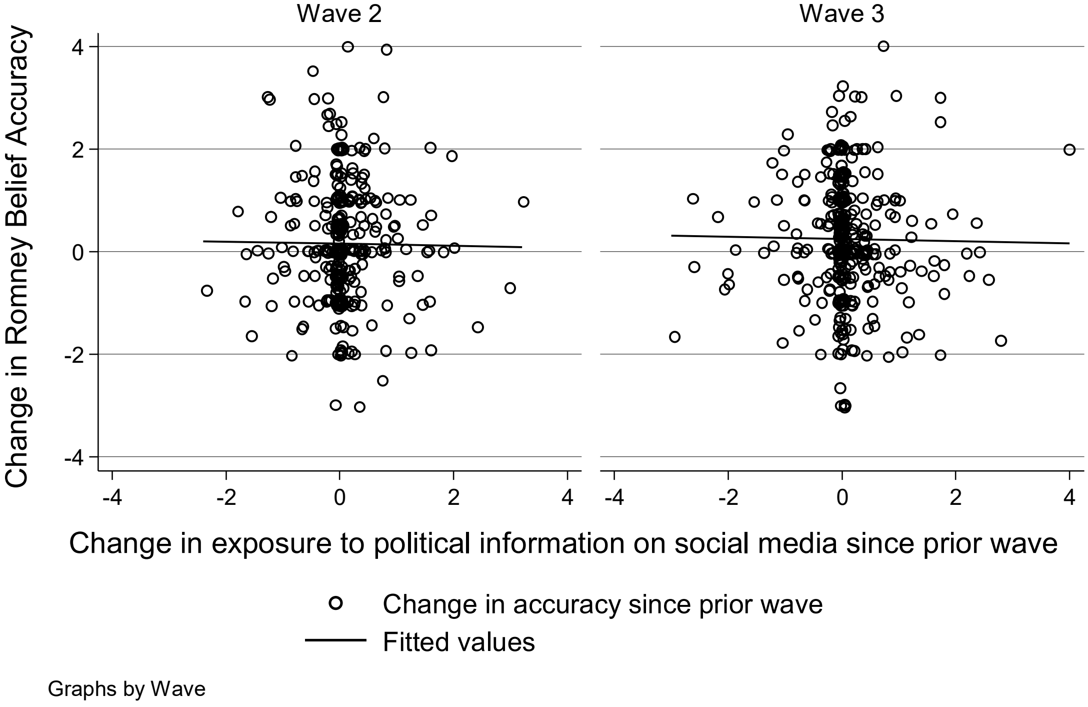

Note. Top figure (1) shows cross-sectional relationship, bottom figure (2) shows relationship between changes in social media use and belief accuracy.

Fig D. Wave-over-wave change in social media use, 2016

(1)

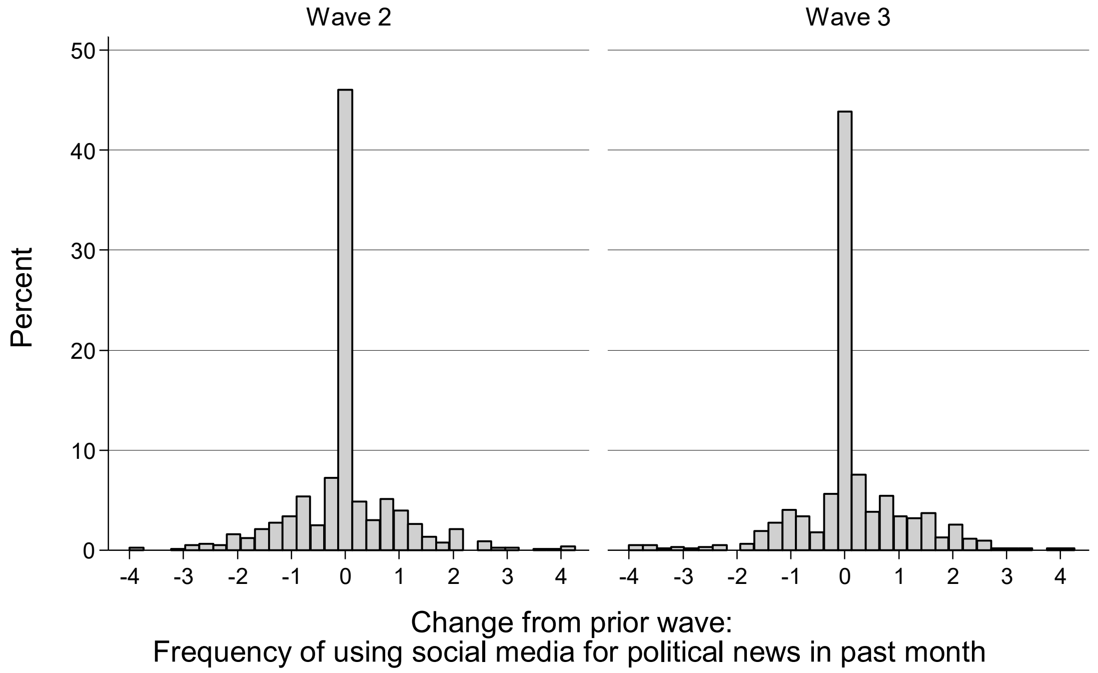

(2)

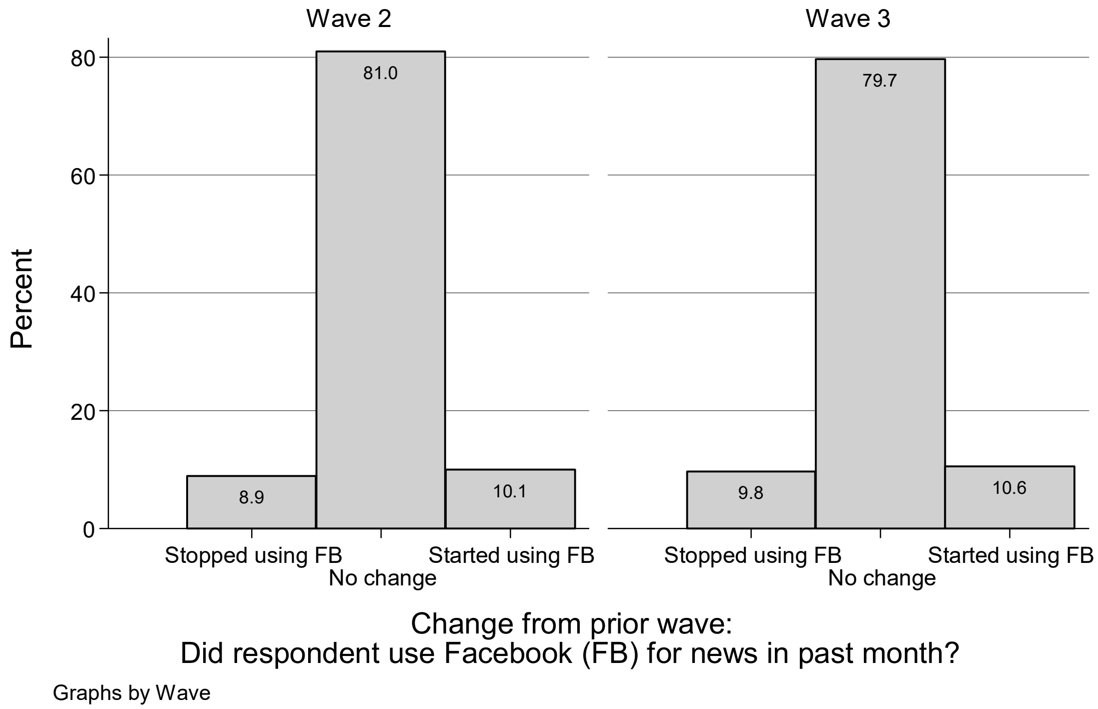

Note. Top figure (1) shows distributions of change in frequency of social media use from prior wave in 2016. Bottom figure (2) shows the distribution of respondents who stopped, started, or continued using Facebook since the prior wave.

Fig E. Scatterplots comparing social media use and campaign issue accuracy.

(1)

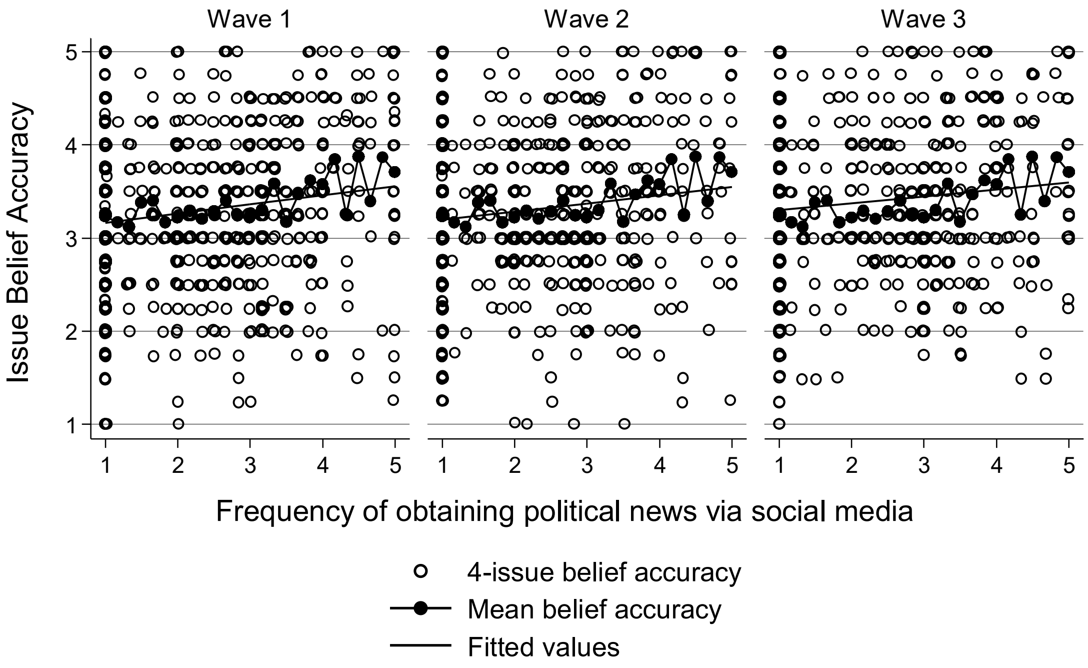

(2)

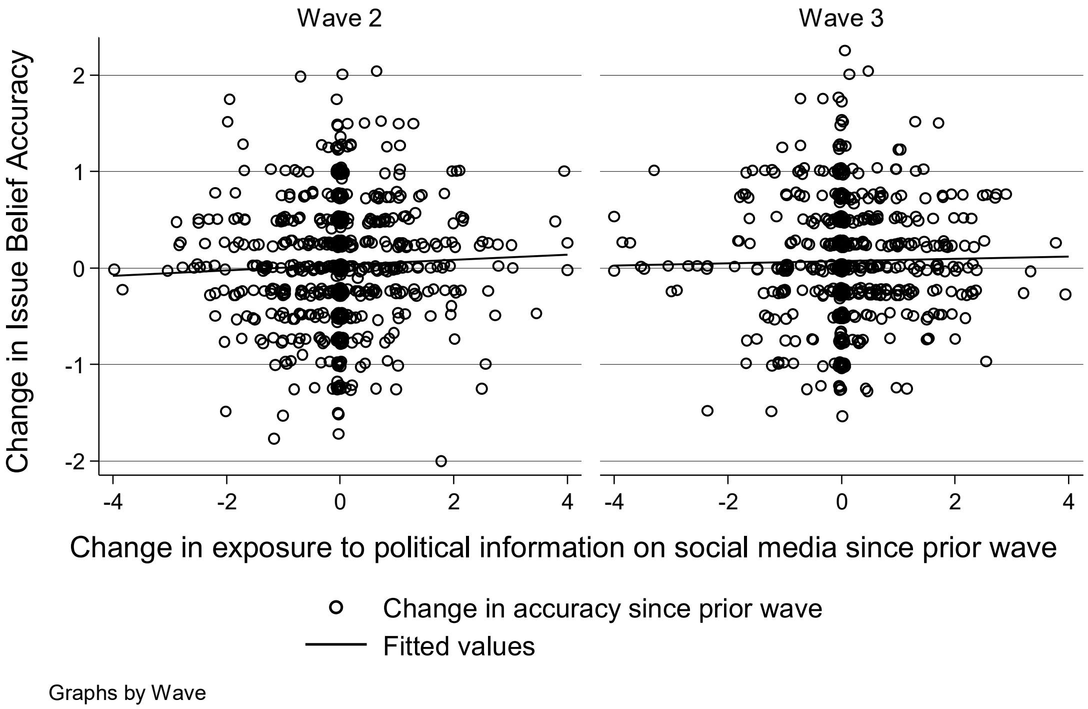

Note. Top figure (1) shows cross-sectional relationship, bottom figure (2) shows relationship between changes in social media use and belief accuracy.
